# The dynamics of sexual risk amongst South African youth in age-disparate relationships

**DOI:** 10.3389/frph.2023.1125552

**Published:** 2023-07-18

**Authors:** Phiwokazi Qoza, Alastair van Heerden, Zaynab Essack

**Affiliations:** ^1^ Centre for Community Based Research, Human Sciences Research Council, Pietermaritzburg, South Africa; ^2^ SAMRC/WITS Developmental Pathways for Health Research Unit, Department of Paediatrics, School of Clinical Medicine, Faculty of Health Sciences, University of the Witwatersrand, Johannesburg, South Africa; ^3^ School of Law, University of KwaZulu-Natal, Pietermaritzburg, South Africa

**Keywords:** sexual risk behaviour, sexual risk perception, social capital, IPA, seroguessing, age-disparate relationships

## Abstract

**Introduction:**

South Africa has the highest burden of HIV in the world with over 8 million people living with HIV. Young women and girls account for a quarter of new HIV infections while making up only 10% of the population. A key driver of HIV incidence is transactional and survival sex between adolescent girls or young women and older men (the latter referred to as ‘sugar-daddies’ or ‘blessers’). This paper expands on the existing literature on age-disparate and intergenerational relationships to provide social and behavioural interpretations of how young men, commonly omitted from studies on this topic, and women in concurrent relationships with both their peers and older partners perceive and navigate sexual risk.

**Method:**

We conducted a qualitative study in a rural setting of uMgungundlovu District, KwaZulu-Natal Province, with purposively selected male and female participants aged 18–24 years old in age-disparate relationships. Semi-structured in-depth interviews (IDI) were conducted and analysed using interpretative phenomenological analysis (IPA) to explore existing information, motivations, and behavioural practices around relationships and sexual risk.

**Results:**

The themes and related sub-themes found through IPA included the following: (1) navigating dating: narratives that show a strong preference for being in an age-disparate relationship; the challenges that young people face when choosing an older person as a side partner; and social media applications seen as creating opportunities to meet side partners; and (2) the distribution of love and trust in a multi-party sexual network: condom (mis)use differentiates between straights—those in a serious relationship—and sides; and the power of eye-test seroguessing, the praxis of testing people visually for HIV in nullifying existing knowledge about sexual and reproductive health risk.

**Discussion:**

This research offers an understanding of how schemas of non-condom use are organised. We observed that while condom-less sex is often viewed as essential to building social capital in a serious relationship, it is not the only factor that determines sexual relationship power. Eye-test seroguessing not only develops consortium (trust, reciprocity, and solidarity), but it fulfils the psycho-social need to belong to a network of serious relationships. Moreover, it is critical to the enactment of masculinities because it consolidates femininity to keep men happy, i.e., by being passive in the sexual encounter, women constrain their self-efficacy to act contrary to the conventions of reputable women. Therefore, it is plausible that in the serosorting that occurs prior to unprotected sexual acts, the power of eye-test seroguessing limits the ability to engage in safe sexual practices.

## 1. Introduction

An estimated 5.25 million South Africans were living with HIV in 2010, a number which increased to 8.2 million by 2022 (1, 2). Black African females have and continue to constitute the majority of people living with HIV (PWH) globally, and HIV incidence in this group is significantly higher than that of their male peers (3–6). As a result, HIV association literature has emerged to interpret how individual choices contribute to collective risk behaviours fuelling gender-disparate HIV prevalence, treatment, and care outcomes between men and women. There are a number of biological, behavioural, social, and structural factors associated with earlier HIV acquisition amongst women, including participation in survival sex and transactional sex with partners between 5 and 10 years older, and sexual relationships with ‘blessers’ (4, 6–10). In a manoeuvre to posit women who engage in transactional sex as powerful agents and not mere victims of racialised and gendered poverty, the literature transcends from a narrative of ‘real’ deprivation to focussing on a sequence of acts that illustrate agency. Following which, it has been established that many adolescent girls and young women engage in age-discordant transactional sex relationships for more than basic economic needs, instead seeking patrons who will provide them with the latest smartphones, expensive hair weaves, and ‘baecations’^1^ —amongst other things—to feel included in globalised consumer culture femininity portrayed or desired by their peers or on social media (11). Whether they are viewed as victims of economic disadvantage or strategically positioning themselves for upward mobility, it is often suggested that age-disparate relationships are prone to sexual risk behaviours, such as inconsistent condom use with concurrent partners, due to economic and gender-based power structures common in patriarchal societies.

Although HIV incidence has declined in some of African countries between 2005 and 2015, this marked decline has not been observed in the rural KwaZulu-Natal (12). Instead, HIV incidence is not only increasing for young men and boys, but the mean age at infection is declining (5, 13). Others have long argued that gender disparities in HIV incidence and prevalence are merely signs of delayed testing amongst young men and boys. In the alternative, young girls have been shown to test at higher rates due to frequent visits to healthcare facilities—to collect contraceptives for instance (14, 15). Age-disparate relationships also occur between older women and younger men, but have rarely been associated with HIV transmission (16). There is a growing body of literature that posits that the HIV risk profile of middle-aged women who engage in sexual relationships with younger men is higher (17, 18). Moreover, younger men in sexual relationships with older women presume their partners have the financial resources to take care of their health, thus minimising their likelihood of transmitting sexual infections/diseases (19).

The response to the HIV epidemic is in want of a shift in perspectives that view HIV testing as a last resort to confirm morbidity rather than as a measure to periodically ascertain sexual and reproductive health. There persists a strong association between HIV testing and negative affects such as anxiety, fear, and shame (20). It is common to delay testing until a symptomatic diagnosis, i.e., the stage in which one is displaying a range of symptoms associated with low CD4 count (e.g., swollen lymph nodes, poor appetite, high fever) (21). For many, it is better to not know their HIV serostatus if they are not experiencing such symptoms. Another avoidance technique, which has been employed by men who have sex with men, is to intentionally seek sexual partners that they assume to be their preferred serostatus. Whereas the criteria may differ from person to person, Kuhn et al. (22) mention physical attractiveness and non-insistence on condom use, amongst others, as what some view as signs of sero-negative status. Zablotska et al. (23), however, are doubtful whether serosorting is an effective risk reduction strategy for individuals who do not test together. They posited that, in the absence of status disclosure and validation through testing, such techniques should more appropriately be referred to as seroguessing because they assume seroconcordance.

The extent to which heterosexual partners seroguess prior to engaging in sexual activity remains underexplored in sub-Saharan Africa. This research seeks to build an understanding of how young people perceive sexual and reproductive health risks in their romantic and sexual relationships in the era of poverty, socio-economic inequality, HIV, and universal test and treat.

## 2. Method

This research took place in a rural setting in uMgungundlovu District in KwaZulu-Natal Province, which reports the highest provincial HIV prevalence, estimated to be between 20% and 30% of those above 16 years old, in South Africa (24). None of the participants, who were purposively recruited for in-depth interviews (IDIs), were in formal employment at the time of the interviews. The eligibility criteria included that individuals be between 18 and 24 years of age, and report being in a sexual relationship with at least one person in the past 18 months who was 5 years or older than themselves. The interview questions were designed to solicit the narratives, opinions, and experiences of decision-making around HIV testing, condom use, contraceptive use, pregnancy, and related topics. The interviews were conducted by locally hired and trained female data collectors. All interviews were conducted in the IsiZulu language and were audio-recorded, translated, and transcribed to English.

The data were analysed using interpretative phenomenological analysis (IPA) techniques. IPA seeks to understand the perspectives of candidates by gathering ‘the meanings which such people make of life’ (25). The first stage of the analysis entailed ‘active engagement with the data’ (25). During this stage, we did an initial reading of the scripts and listened to the audio files to experience the journey that the participants used to make meaning. This was followed by initial coding; ‘the making of descriptive, linguistic, and conceptual comments deduced from the interview data—both verbal and linguistic’ (25). The coding involved exploring the verbatim descriptions of the participant to understand the ‘functional aspects of language such as pauses, laughter, tone, repetition, and metaphors’ and ‘identify key phrases, explanations, descriptions, and emotional responses’ (26). This engagement with the data was iterative as we ‘moved back and forth through a range of different ways of thinking about the data, rather than completing each step, one after the other’ (25). After a number of attempts to make sense of the commonalities between the verbatim statements, most of the perspectives of the participants were organised in ways that critique, contribute to, and elevate a number of conceptual and theoretical frameworks that seek to interpret lived experience.

The coding was supported by the ATLAS.ti software.

## 3. Results

A total of 17 participants were included, 11 females and 6 males (Table 1). The median age of the participants was 21 years (interquartile range: 19–23). Two broad thematic areas and related sub-themes emerged: (1) navigating dating: narratives that show a strong preference for being in an age-disparate relationship; the challenges faced when choosing a blesser as a side partner; and social media applications seen as creating opportunities to meet side partners; and (2) the distribution of love and trust in a multi-party sexual network: condom (mis)use differentiates between straights and sides; and the power of eye-test seroguessing in nullifying existing knowledge about sexual and reproductive health risk.

**Table 1:** The participants demographics.

		Frequency (*N* = 17)	%
Race	Black African	17	100
Age	Median (IQR)	21 (19–23)	
Sex	Male	6	35.3
	Female	11	64.7
Education level	None	2	11.8
	High school	14	82.4
	Tertiary education	1	5.9
Own mobile device	Yes	17	100

### 3.1. Navigating dating

None of the participants in this study were in monogamous relationships, often engaging in sexual intercourse with people closer to their age and older individuals. They stated that peer relationships are becoming rare as younger people are seeking, and are being pursued, by older people. They typically differentiated between their partners by referring to the primary partners as ‘straights–mains’ and other partners as ‘sides’. For some female participants, the older partners or ‘blessers’ were their ‘sides’ with whom they did not anticipate long-term relationships. Often, the blesser served the purpose of fulfilling financial wants and needs that the primary partners could not meet. The opportunity, however, to meet side partners on social media applications often created conflicts in straights–main relationships.

#### 3.1.1. There is a strong preference to be in age-disparate relationships

When asked to paint a picture of the dating scene, many young people viewed peer relationships as becoming rare for people their age, irrespective of gender.

These days it's very rare to find people of the same age group who are in a relationship… But that is my opinion. Girls date older men and boys date older women—Female, 22 years old

I think that because we are the same age, that's why things don't always work out. It's rare in most cases to find people that are the same age dating. It's rare—Male, 24 years old

The participants expressed less stress, financial benefits, access to materials things, freedom of movement, and flexibility of time as some of the benefits of age-disparate relationships.

You see… you get more stressed if you are dating someone your age group because they won't be able to take care of you and your responsibilities. The only thing they will do is have sex with you and also go have sex with someone else, then you will get more stressed thinking about the fact that they kept having sex with you over and over again—Female, 23 years old

They buy us clothes or give you money something like that; there is a lot. But in most cases, it is money. It also depends on what they have—Male, 18 years old

They like cars so they date older women so that that they can drive their cars and go check other girlfriends with that car—Female, 23 years old

…. they [older men] are better, like they can call you in the morning, afternoon and in the evening. Younger people only send you WhatsApp and they only make their friends happy, posting statuses, and wearing nice clothes and that is it—Female, 19 years old

Unlike younger women who have excuses, an older woman will never tell you that she cannot see you because it is late and her parents do not want her to be out late or they are busy with school work. Whereas the older person if they are at work, they will let you know when they finish work—Male, 20 years old

Many of the participants expressed that their decision to be in an age-disparate relationship was influenced by their peers and a desire for financial or material benefits that enhance their social status (real or perceived).

In my age group they encourage you to date an older person—Male, 23 years old

My friends would encourage me to date the old man, they would say ‘take him friend and chow his money, these old men have diabetes anyway so he won’t have sex with you’—Female, 23 years old

because you see the gifts he gets from his date. And the date will come in her car and they will park somewhere, then you feel the pressure of also wishing to date an older person—Male, 18 years old

There are those who have influence, especially when you see them driving a very beautiful car, or you hear that so and so is getting married, and is marrying an older man. That is what leads you to say that you do not want to date anyone young because all they ever know is wanting sex from you, they are not prepared to get married—Female, 23 years old

#### 3.1.2. Challenges faced when selecting a blesser as a side partner

Whereas age-disparate partnerships were desired, and often sought out, some women in age-disparate relationships distinguished between older partners and blessers. For example, the participants rarely referred to their older partners as blessers—despite popular use of the term in the South African context.

Blessers are people who are married. Someone who is very old…. Someone old enough to be my father. I would not call someone who is 5 years older than me a blesser. A blesser is someone I would date because I wanted a new hairstyle. Or I wanted new clothes. Someone who can do things for me. I can date someone who is 5 years older than me but I would not view them as a blesser—Female, 22 years old

I [started] dating someone who is a bit older who is 33 years… he cares about me, he treats me well and he also respects me. He is a taxi driver—Female, 20 years old

I am 24 years old but I'm dating someone who is older than me by 7 years which means that the person is 31 years old. What I've seen is that an older partner gives you time… also when it comes to certain basic needs—Female

Blessers were often framed as older side partners and a means to access benefits that the primary partners could not provide, for example:

Sometimes you have someone who you love, but they cannot give you stuff so you get someone else who can give you things on the side. Sometimes the side guy has a car so he can take you places. Sometimes he is a taxi driver so you won't have to pay taxi fare. But you have someone you love while having these other side relationships—Female, 20 years old

… you find that a straight partner is unable provide for your needs, then you resort on having another partner who will be able to provide for your needs—Male, 23 years old

 … they opt for having multiple partners, the other one meets the financial needs whilst the other one is there for the individual emotionally and they find that they become happier that way—Female, 22 years old

The above extracts underscore that young people rationalise multiple partnerships as required to fulfil different desires of love, emotional well-being, and financial benefit.

Moreover, there were many narratives that challenged the desirability of the blessers.

For me if you are an older man dating a younger girl you are taking an advantage—Male, 24 years old

They take advantage because they are older and you do not know anything because you are young. When you are young, you go crazy when someone tells they love you—Female, 22 years old

 …Especially older people in most cases will want to have sex without using a condom and this will force you to do things that are against your will. The older partner when you suggest that they get tested for HIV and such, they are quick to say that they are older and will do no such a thing—Female, 22 years old

People are scared of dating blessers these days because some use *muti* (using traditional medicine to have spiritual powers through rituals and totems) to get money and they want to use that *muti* with you. You go to their house and they tell you that you should do some things (with their totems) beforehand so those things are scary—Female, 19 years old

Older people overpower us because we are young and they do things for us. It depends… I can’t say all of them are like that, but the ones I know *hayi!*. And another thing is you cannot freely communicate everything with them. Even if I think he has done something wrong… it's difficult to have those conversations with him because he does things for me and is older than me. So out of respect, and fear that he might beat me, I am unable to communicate many things with him—Female, 20 years old

Older people are evil because they see someone like me who is young and decide to pursue a relationship. Someone who could be their child? They are taking advantage. Advantage to beat you…—Female, 19 years old

The quotes above highlight the inherent power dynamics in intergenerational relationships with both male and female participants perceiving that older men ‘take advantage’ of younger women or exert power over women through ritualistic practices. The participants are silent about the roles of women in initiating these relationships but frame the older men as predatory in this dynamic, e.g., ‘older people are evil’. The common rhetoric across the participants is of being vulnerable or taken advantage of. This perspective lives in parallel to some narratives that blessers are intentionally sought for financial benefits.

#### 3.1.3. Social media applications seen as creating opportunities to meet side partners

As many young individuals cannot meet all the needs of their similarly aged partners, and know their partners may seek their needs to be met through side partners, many monitor the social media behaviour of their partners. The participants often accused their partners of using their social media posts and reactions to attract suitors. Whereas, some state that virtual romances never end in meet-ups with ‘Facebook’ friends or ‘WhatsApp’ contacts, some have transcended to physical space interactions.

…on WhatsApp, especially if we fought. he would hide everyone and post a girl so that I would see and be angry, but I don't care about that, and I always tell him that I don't care that he is older than me and what he is doing I can do it better than him so that he would feel what he is doing to me. If we broke up, I would post a picture of a man that I don't even know so that he would be angry [laughs]—Female, 21 years old

I usually fight a lot with the father of my child because of the things he posts on his statuses. He would reprimand me about some pictures that I post, he is quiet, and strict although he is younger. For example, this one time I posted a picture of myself wearing leggings or a short skirt, he would say I should remove those pictures and ask if I am selling my body because of the pictures I'm posting—Female, 23 years old

In these relationships, social media played a significant role as a space to find suitors or in vengeful responses to challenges in their main relationship. Moreover, it seemed difficult for those who consider themselves as straight partners to ignore the omnipresence of virtual connections. Their discovery by straight–main often led to emotional and physical abuse;

Yes, so for example, if the father of my child posts something on Facebook you will find girls who would call him ‘baby’, then we would fight over that because I would ask him about it and tell him to delete that comment. Sometimes he would say that he doesn't even know that person. I would tell him to tell his friends on Facebook that he has a child with someone and I don't like the things they say—Female, 22 years old

In most case we will fight over the issue of phone, because she had her ex-boyfriend, I advised her to change her numbers. Her sisters were friends with her ex-boyfriend so, they will give him the new number maybe they will meet in town and he will ask for her number, and they will give him. So, we will fight over it because I didn't know how he got hold of the new number and we will fight over that issue. Because she had psychotic trait, she will smash the phone on the wall. We used to fight a lot over the issue of the phone—Male, 20 years old

It follows that young people in relationships are using social media to negotiate power within relationships. Some use social media as surveillance of the fidelity of their partner, enquiring about the times their partner is ‘on-line’, demanding to read the messages of their partner, and occasionally ask their partners to delete the posts. Others use this information to physically assert their position in the relationship. However, most did not initiate break-ups upon discovery of deceit by their straight–main, but rather pursued another relationship, concurrently, with someone else.

### 3.2. The distribution of love and trust in a multi-party sexual network

While young men and women who have been dating for a significant period often stop using condoms to illustrate that they love and trust their partner, the participants admitted that they do not regularly test during concurrent relationships to validate their HIV status. Instead, they may take temporal breaks in the straight–main relationship to entertain other people on social media or real life. Following which, condom use can be inconsistent in both the straight­–main relationship and side relationships. An example of why this becomes a sexual and reproductive risk behaviour is illustrated by the power of eye-test seroguessing, which can nullify existing knowledge about sexual and reproductive health risk.

#### 3.2.1. Condom (mis)use can differentiate between straights and sides

Most participants had multiple sexual partners at the time of the interviews.

[My boyfriend]… was cheating on me while in (Suburb) and I am here in (Suburb). So I also started cheating here in (Suburb)—Female, 23 years old

‘*Thina singabafana siye sigange*’—we are boys, we sometimes do naughty things (implying pursuing other people while in a relationship)—Male, 20 years old

Despite knowing why condom use was important, many participants do not use condoms to illustrate that they love and trust their main partner(s).

The person is able to choose whether they use a condom or not. Maybe with the person they've had the longest relationship with they were not prone to using protection all the time—Female, 24 years old

I trusted her because whenever I would go to her place she was there so we stopped using condoms—Male, 23 years old

You only use condom on other partners since they are not your straight partner. That is how you decide you don't use it on your straight partner because you trust her—Male, 21 years old

Although some admit to having tested with their straight–main partners at the start of the relationship, it is unclear whether they continue to validate HIV status as more individuals are introduced into their sexual network.

‘Sometimes it happens that you have a straight partner (primary partner) and you will focus on me then they will disappoint you. When they have disappointed you that when you decide that I might as well cheat on him because he has disappointed me‘—Female, 18 years old

‘So, I decided to date this guy because my main partner was cheating and I found out that he was cheating and I'm not a confrontational person… I started dating this person because I wanted to get back at my main partner‘—Female, 21 years old

What can be deduced is that introducing condoms and regular HIV testing would illustrate a lack of trust, damaging the social capital that the straight–main relationship is embedded on.

‘I think it's better if we buy and she sees that there was no condom (at the time of initiating sex). Because even if she saw them and my friends come and ask for condoms, and I give him, she will come back and see that the condoms are open and the problem would start’—Male, 23 years old

‘Condoms cause fights. One of my partners would count the condoms and although I had used them with her, she would fight me about that’—Male, 18 years old

The participants did believe that HIV testing was important in the face of real or imagined infidelity. However, some were not getting tested with all their partners, and thus could not guarantee their status. For instance, it was common for individuals to state that they were testing for HIV separately;

‘If you have a straight partner, you would both go to the clinic or doctor and test for HIV, and even if your straight partner stays far way from you, you would decide to go to the clinic separately, but whenever you meet you would show each other HIV test result’—Female, 23 years old

‘Sometimes you will go to the clinic for HIV test and sort of get someone else to get the results using their details. When I found out that my baby daddy was hiding certain things from me…he asked his friend to an HIV test and write down his details and showed me that he was HIV negative whereas he was positive. When I suggested that we get tested together, he would sort of push that conversation aside. Right after my pregnancy I found out that he had fraudulent HIV result all along because when you are pregnant you need to go for all those checkups’—Female, 24 years old

‘…we did talk about those things, but we never tested together. We test separately’—Female, 19 years old

These narratives illustrate how a sexual network characterised by uncertain seroconcordance transpires. Some participants do not test for HIV together at all and others admit to testing at the start of the relationship only. In the latter, it is unclear whether individuals continue to test together as more individuals are introduced into their sexual network. What is common cause is that individuals are unlikely to insist on condom use as this can create conflict in their relationships.

#### 3.2.2. The primacy of eye-test seroguessing

It was common for the male participants to either knowingly initiate sex without being in possession of any condoms or to realise in the process of initiating sexual intercourse that they had no condoms. To resolve the ‘condom conundrum’, many merely conducted non-verbal serosorting to enable them to continue with unprotected intercourse. They do this by testing people visually to ascertain whether the individual is ‘likely’ to have HIV.

‘There are girls when you look at them, you feel that this is not a type you can use the condom on her’—Male, 20 years old

‘I can judge a person by her physical appearance, then I can make conclusion that she is fresh, has nothing and everything is alright with her’—Male, 23 years old

‘I am dating several partners… one is 24 years old. She is fit. I mean physically well-built… so we do not use a condom’—Male, 18 years old

The notion of physical appearance as a precursor to virtuous behaviour was also observed in the way younger men spoke about their older partners. For instance,

‘I have not tested with my side partner who is 27 years old. I do not know her status. I like that she takes care of herself and is classy’—Male, 23 years old

‘I practice the pull-out method so I know she will not get pregnant. She is 6 years older than me…. She is beautiful, she is beautiful [laughs] her beauty was the first thing that attracted me [laughs]’—Male, 21 years old

In this study, epithets such as ‘fresh’ or fit body shape or figure were linguistically framed as markers of an HIV negative status. It follows that some young men will *never* use condoms if they find the woman they are courting attractive. This is irrespective of whether they view the woman as a straight partner, a side partner, or a one-time partner. In addition, if condoms are not used from the start of the relationship, there is little to no motivation to introduce condoms as the relationship progresses because the participants continue to guess HIV status based on what they believe their eyes are showing them.

## 4. Discussion

This study explored decision making amongst young people in multi-party sexual networks with both age-disparate partners and partners closer to their age. Although the participants expressed a strong preference to be in age-disparate relationships, the young women struggled to balance being in concurrent relationships with straights, or primary partners, and blessers. This is illustrated by how young women emphasised the growing undesirability of blessers, framing them as individuals who were taking advantage of young people or individuals who could not meet the romanticised ideal of blesser–blessee relationships. This shift in attitudes, a rejection of transactional sexual relationships between younger women and older men, has been noted before. For instance, in Hlabangane (27), blessers were said to be growing weary of the susceptibility of young women to contract HIV to the extent that it would be rare for a blesser to insist on having condom-less sex with blessees. The young women, however, did not express the same level of scrutiny to and suspicion for the young men they also enter into sexual relationships with. This is significant because most young men do not view being in sexual relationships with multiple individuals as conflicting because it is culturally acceptable for them to pursue, and in some cases, marry multiple partners. For our participants, selecting a side partner and deciding whether to introduce condoms into the relationship was based on whether young men found the person attractive or not. Whereas some stated that the absence of condoms during sex is reserved for the people they love and trust; this becomes unstable as they eventually stop using condoms with everyone regardless of the nature of their relationship.

The cultural scripts of what makes an eligible bachelor imply that poor boys and young men should not be able, and are unable, to keep multiple sexual partners. Similarly, as some older women do not have the physical attributes associated with youth, they should not be able to attract and keep young sexual partners. Doomed from the start as they lack cultural legitimacy, age-disparate relationships between older women and younger men are believed to occur because older women have little to no expectations of long-term commitment from younger men that suits young men who are unwilling to offer long-term commitment (28, 29). What emerged in this study is that participation in age-disparate relationships by older women and younger men enables both of them to establish ‘vertical points in the social hierarchy’ that tips the balance of power in their favour (30). Once provided with financial benefits or access to material things, young men can appear as a man to their families and younger girlfriends. For instance, the participants often underscored that young men in intergenerational relationships can have access to the material possessions of older women, such as their car, which most young men use to visit other women they are also in relationships with.

The likes of Mgwaba and Maharaj (31) have characterised the existence of multi-party sexual networks as being about *ukujola* whereupon men expect immediate sexual gratification, *in lieu* of being ridiculed, by their peers, for planning ahead by keeping condoms or waiting for a future uncertain date to consummate the relationship. Whereas earlier literature claimed that men do not like to use condoms because they do not enjoy sex with condoms; through condom-less sex with women they consider attractive, young men are able to gain prestige amongst their peers, ‘a homosocial status derived from sleeping with high-status women’, that consolidates masculinity (27, 32). It could, therefore, be argued that the ability to make the will of women pliable enough to consent to unprotected sex is a schema that is critical to the enactment of masculinities. That is, the performance of masculinity relies on the cultural scripts about the significance of unprotected sex to convince a stranger that through this one act they, too, occupy the same position as a straight–main. The process of ‘winning’ women over also forces young women to consolidate their femininity to ways that keep men happy, i.e., by being passive in the sexual encounter, women constrain their self-efficacy to act contrary to the conventions of reputable women.

All relationships produce and are a product of social capital, a network of ties fuelled by norms of solidarity, reciprocity, and trust [Bourdieu in (33, 34)]. In the networks of ‘straight–main’ ties and or ‘side’ ties, each engagement exists through a structure that supposes and reinforces norms and rules about ‘the strength and centrality of the tie’ (35). Many assume seroconcordance because they seek to belong to the network of straights with the men courting them instead of calculating sexual and reproductive risk that can accrue from their involvement in a multi-party sexual network. There are many schemas employed to join and stay within the network. One that was common for the participants in this study was to rely on eye-test seroguessing to avoid ‘fighting’ about condoms, testing, and HIV status. This may also be a schema employed to negate acknowledging that if there was a foundation of trust, conversations about condoms, HIV status, and testing would not break down the centrality and density of the tie. Therefore, this also implies that eye-test seroguessing fulfils psycho-social needs for—despite issues of mistrust and the potential of sides—many of the women in this study prefer being in the network of straights than being in no relationship or in a transient relationship with a blesser. In assuming seroconcordance, however, there might be a high degree of serodiscordance, as well (23). This research contributes to the existing research on seroguessing, which has often focused on men who have sex with men in the Global North. In this rural sample of heterosexual South African participants, they seroguessed during the recruitment of new sexual partners and also in longer-term relationships who have never introduced condoms, who have stopped using condoms, and who do not test together as well.

Although it might appear that there were no consequences to cheating in straight–main relationships because the young women stayed in them irrespective of concurrent dating, all individuals expressed that infidelity created unhealthy communication, followed by verbal and, in some cases, physical abuse. Many of the young men and women stated that cell phone and or a social media use made them fight with their partners. Implicit in their behaviour on social media or their responses to social media posts and reactions is an attempt to negotiate power in their relationships. The type of digital intimate partner violence, where one partner influences or manipulates another to curate their inputs according to their desires, poses a threat to the overall well-being of young people and their perceptions of relationships in the long term. Due to several factors that lead to distrust, such as social media posts and the absence of consequences for cheating, having sex without a condom might be the only way these young people feel that they can ensure their partners love them. Many participants often presented this sexual risk as a conflict resolution mechanism because condoms cause fights. Since social capital is created during unprotected sexual acts, it is plausible to suggest that during the serosorting that occurs prior, the power of eye test seroguessing has the ability to nullify existing knowledge about sexual and reproductive health risk. Moreover, it is likely that eye test seroguessing plays a key role in perpetuating the HIV epidemic, and preventing the achievement of global HIV prevention targets, in South Africa.

### 4.1. Recommendations

These findings should be explored further in larger scale studies given the critical finding that seroguessing is used to manage condom use by young people, in relationships with peers and older persons. This risky sexual practice is amplified in the concurrent relationships described by the participants in this study and is worth further exploration. The findings call attention to the need for improved awareness that HIV testing is the only way to determine HIV status, and the value of prevention methods like pre-exposure prophylaxis when engaging in risky sexual practices and/or there is uncertainty about the HIV status of a partner. Despite the prominence of life orientation sexual and reproductive health education in South Africa during high school (36), in a context where a large majority of youth who have recently finished high school are unemployed, many do not have opportunities to access information about risk assessment into adulthood. Sexual health education interventions for young adults are needed to create awareness about the evolving dynamics of sexual relationships into adulthood. Moreover, there is a need to cultivate a shift in the perspectives of many young people who view HIV testing as something to be avoided. Young people need to be motivated to view HIV testing as *a priori* to commencing a sexual relationship instead of assuming seroconcordance based on visual assessments.

### 4.2. Limitations

The participants were interviewed in IsiZulu and the transcripts were translated and transcribed into English. This potentially limits the depth of the data as a lot of meaning might be lost in the process of translation.

## Data Availability

The raw data supporting the conclusions of this article will be made available by the authors, without undue reservation.

## Footnotes

^1^A vacation or weekend getaway, by a couple or individuals who are dating, to a revered or secluded location.
